# Cost-effectiveness of Telaprevir Combination Therapy for Chronic Hepatitis C

**DOI:** 10.1371/journal.pone.0090295

**Published:** 2014-03-06

**Authors:** Anita J. Brogan, Sandra E. Talbird, James R. Thompson, Jeffrey D. Miller, Jaime Rubin, Baris Deniz

**Affiliations:** 1 RTI Health Solutions, Research Triangle Park, North Carolina, United States of America; 2 RTI Health Solutions, Waltham, Massachusetts, United States of America; 3 Vertex Pharmaceuticals Incorporated, Cambridge, Massachusetts, United States of America; Hopital Bichat Claude Bernard, France

## Abstract

**Objective:**

To explore the expected long-term health and economic outcomes of telaprevir (TVR) plus peginterferon alfa-2a and ribavirin (PR), a regimen that demonstrated substantially increased sustained virologic response (SVR) compared with PR alone in adults with chronic genotype 1 hepatitis C virus (HCV) and compensated liver disease in the Phase III studies ADVANCE (treatment-naïve patients) and REALIZE (relapsers, partial responders, and null responders to previous PR treatment).

**Study Design:**

A decision-analytic model was developed to assess the cost-effectiveness of TVR+PR vs. PR in the United States (US).

**Methods:**

Patients first moved through the 72-week decision-tree treatment phase of the model and then entered the cyclic Markov post-treatment phase. Clinical data (patient characteristics, SVR rates, and adverse event rates and durations) were obtained from ADVANCE and REALIZE. Health-state transition probabilities, drug and other costs (in 2012/2013 US dollars), and utility values were obtained from the trials, published studies, and publicly available sources. Outcomes were discounted at 3% per year.

**Results:**

Regardless of treatment history, patients receiving TVR+PR were projected to experience fewer liver-disease complications, more life-years, and more quality-adjusted life-years (QALYs) than patients receiving PR. In prior relapsers, TVR+PR was dominant, with lower total medical costs and more QALYs. For the other patient subgroups, incremental costs per QALY gained were between $16,778 (treatment-naïve patients) and $34,279 (prior null responders). Extensive sensitivity analyses confirmed robust model results.

**Conclusions:**

At standard willingness-to-pay thresholds, TVR+PR represents a cost-effective treatment option compared with PR alone for patients with chronic genotype 1 HCV and compensated liver disease in the US. Future analyses are needed to compare TVR+PR with all existing HCV treatment options.

## Introduction

Of the worldwide population, 2% to 3% (130–170 million people), including approximately 3.2 million in the United States (US), are chronically infected with the hepatitis C virus (HCV) [1], [2]. While incident cases have declined dramatically in the US over the past two decades [3], estimates of prevalent cases of chronic HCV infection have remained stable, primarily due to high rates of infection in earlier decades and the chronic nature of the condition [2], [4]. Although HCV infection progresses slowly, it can eventually lead to scarring of the liver (i.e., cirrhosis); progression toward liver failure, including decompensated cirrhosis (DCC) and/or hepatocellular carcinoma (HCC); and premature death [4]–[6]. As individuals with HCV infection age and progress, HCV-related complications and deaths are expected to continue to increase [4] and associated medical-care costs are projected to peak later this decade at over $1 billion annually [7].

The primary goal of treatment of chronic HCV infection is sustained virologic response (SVR), defined as undetectable HCV RNA 24 weeks after completion of treatment. Genotype 1 HCV infection accounts for about 75% of all cases in the US and is the most difficult to treat of the six identified genotypes [8], [9]. Among treatment-naïve patients with genotype 1 HCV infection, therapy with a 48-week course of peginterferon and ribavirin (PR) historically has yielded clinical trial SVR rates ranging from 42% to 46% [10], [11]; re-treatment with PR has been associated with lower SVR rates (16.3%) [12].

Telaprevir (TVR) is an HCV NS3/4A protease inhibitor, which in combination with PR is indicated for the treatment of genotype 1 chronic HCV in adults with compensated liver disease, including cirrhosis, who are treatment naïve or who have been previously treated with interferon-based regimens. The efficacy and safety of TVR+PR compared with PR alone were investigated in the Phase III, randomized, double-blinded, multicenter trials ADVANCE (treatment-naïve patients) and REALIZE (previously treated patients) [13], [14]. In both trials, treatment with TVR+PR resulted in significantly higher rates of SVR than PR alone: 79% vs. 46% for treatment-naïve patients, 86% vs. 22% for prior relapsers, 59% vs. 15% for prior partial responders, and 32% vs. 5% for prior null responders [15].

Achievement of SVR in patients with chronic HCV infection treated with PR has been shown to slow or arrest the progression of liver disease and reduce the associated risk of morbidity and mortality [16], [17], [18]. The objective of this health economic assessment was to explore how the improved SVR rates observed with TVR+PR compared with PR alone may lead to long-term, clinically meaningful improvements in liver-disease complications, survival, and quality-adjusted survival, as well as to examine the cost-effectiveness of TVR+PR versus PR alone.

## Methods

### Model Description

**Figure 1:**
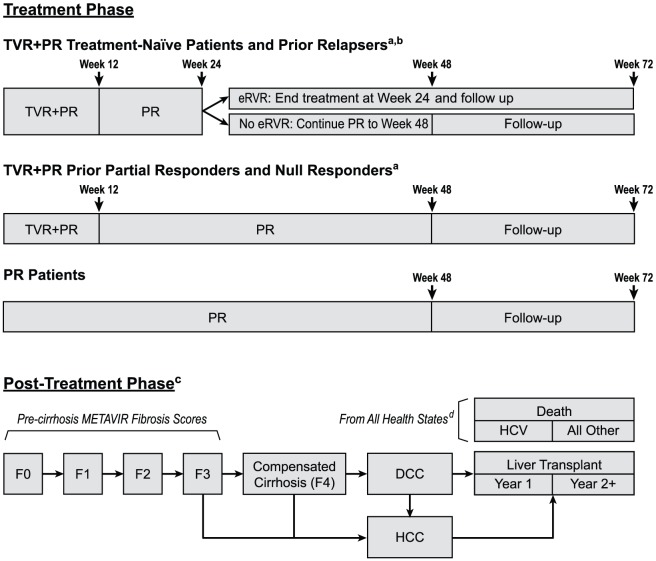
Overview of the Cost-effectiveness Model Structure: Treatment Phase and Post-treatment Phase. DCC indicates decompensated cirrhosis; eRVR, extended rapid virologic response; HCC, hepatocellular carcinoma; HCV, hepatitis C virus; PR, pegylated interferon alfa-2a plus ribavirin; RGT, response-guided therapy; SVR, sustained virologic response; TVR, telaprevir. a Treatment-naïve patients received no prior therapy for HCV, including interferon or peginterferon monotherapy; prior relapsers had HCV RNA undetectable at the end of treatment with peginterferon alfa and ribavirin but HCV RNA detectable within 24 weeks of treatment follow-up; prior partial responders had greater than or equal to a 2-log10 reduction in HCV RNA at week 12, but did not achieve HCV RNA undetectable at the end of treatment with peginterferon alfa and ribavirin; prior null responders had less than a 2-log10 reduction in HCV RNA at week 12 of treatment with peginterferon alfa and ribavirin. b Although not eligible for RGT in REALIZE, prior relapsers were eligible for RGT in the model (i.e., they could discontinue treatment early if eRVR was achieved), per the TVR prescribing information (INCIVEK, 2012) [15]. c Transition probabilities between health states differed depending on achievement of SVR. Specifically, patients with SVR and with no or mild fibrosis (F0–F2) experienced no further liver deterioration. Patients with SVR and with advanced fibrosis (F3–F4) were at continuing risk of liver deterioration, but at lower probabilities than patients without SVR. ^d^ HCV-related death could occur only from health states DCC, HCC, and liver transplant.

A two-phase (treatment and post-treatment) lifetime decision-analytic model was developed in Microsoft Excel to estimate the costs and health outcomes of TVR+PR combination therapy versus PR alone for parallel hypothetical cohorts of patients with chronic genotype 1 HCV infection with baseline METAVIR fibrosis scores F0 through F4 [19], [20]. The population analyzed comprised four subgroups: treatment-naïve patients and patients with relapse, partial response, or null response to a previous course of PR therapy (Figure 1). The model was developed from a US payer perspective, including only direct medical costs.

Patients entered the model at treatment initiation and moved through the treatment and post-treatment phases sequentially. The treatment phase tracked patients for 72 weeks in 4-week time intervals, using a decision-tree structure consistent with prescribing recommendations and with the designs of ADVANCE and REALIZE (Figure 1) [13], [14], [15]. Patients in the PR-alone arm of the model received PR for 48 weeks. Patients in the TVR+PR arm received 12 weeks of TVR with PR followed by PR alone for a total of 24 or 48 weeks of PR. Prior relapsers and treatment-naïve patients who achieved extended rapid virologic response (eRVR) (i.e., undetectable HCV RNA at weeks 4 and 12) received a total of 24 weeks of PR. Prior relapsers and treatment-naïve patients who did not achieve eRVR and all prior partial and null responders received a total of 48 weeks of PR. In all arms, therapy duration was subject to futility rules, and SVR was assessed 24 weeks after completion of therapy. Subsequent treatment for those not achieving SVR was not considered in the model.

After completion of treatment, patients entered the lifetime, cyclic, Markov-process, post-treatment phase of the model at the mean age derived from subgroup analyses of ADVANCE and REALIZE in either the SVR branch or the no-SVR branch, depending on treatment outcome. In any 1-year cycle, patients could remain in or transition between four pre-cirrhosis health states (METAVIR fibrosis scores F0–F3), compensated cirrhosis (METAVIR fibrosis score F4), DCC, HCC, liver transplant, HCV-related death, and non–HCV-related death (Figure 1), where individual transition probabilities differed by SVR status. Patients were assumed to age 1 year at the end of each cycle.

### Model Inputs and Data Sources

Tables 1 and 2 present the default values and sources for the input parameters of the model.

**Table 1:** Input Parameter Values, by Patient Subgroup and Treatment Regimen.^a^

Input Parameter	Treatment-Naïve Patients (n = 724)	Prior Relapsers (n = 354)	Prior Partial Responders (n = 124)	Prior Null Responders (n = 184)
**Baseline distribution of patients with chronic HCV infection**				
Age				
Mean (years)	47	51	51	50
Age <50 years	53%	41%	42%	50%
Age ≥50 years	47%	59%	58%	50%
Sex				
Male	59%	68%	58%	76%
Female	41%	32%	42%	24%
Fibrosis score^b^^,^ c				
METAVIR F0	19%	14%	11%	7%
METAVIR F1	19%	14%	11%	7%
METAVIR F2	41%	29%	29%	29%
METAVIR F3	14%	22%	19%	26%
METAVIR F4	6%	20%	30%	33%
**Treatment efficacy by baseline fibrosis score: SVR rate (number achieving SVR/number in subgroup)** c				
TVR+PR				
F0	85% (57/67)	88% (36/41)	72% (6.5/9)	37%% (3.5/9.5)
F1	85% (57/67)	88% (36/41)	72% (6.5/9)	37% (3.5/9.5)
F2	79% (123/156)	86% (73/85)	79% (23/29)	43% (17/40)
F3	64% (33/52)	86% (53/62)	56% (10/18)	42% (16/38)
F4	71% (15/21)	84% (48/57)	34% (11/32)	14% (7/50)
PR alone				
F0	48% (35.5/73.5)	35% (3.5/10)	0% (0/5)	0% (0/2.5)
F1	48% (35.5/73.5)	35% (3.5/10)	0% (0/5)	0% (0/2.5)
F2	49% (69/141)	28% (5/18)	43% (3/7)	8% (1/13)
F3	35% (18/52)	13% (2/15)	0% (0/5)	0% (0/9)
F4	38% (8/21)	7% (1/15)	20% (1/5)	10% (1/10)
**Severe treatment-related adverse events for treatment-naïve patients and for all previously treated patients combined, incidence; mean duration in weeks** d				
TVR+PR				
Anemia	22.3%; 13.1	22.6%; 24.3		
Fatigue	2.8%; 16.8	N/A		
Headache	N/A	0.8%; 40.2		
Leukopenia	N/A	15.4%; 22.6		
Neutropenia	5.2%; 11.7	19.2%; 16.8		
Rash	3.6%; 3.7	N/A		
PR alone				
Anemia	12.2%; 20.9	9.8%; 16.9		
Fatigue	1.1%; 24.7	N/A		
Headache	N/A	2.3%; 0.2		
Leukopenia	N/A	11.4%; 38.1		
Neutropenia	8.3%; 15.6	12.9%; 18.4		
Rash	0.3%; 7.4	N/A		
**Average utility values for 72-week treatment phase** e				
TVR+PR	0.87	0.87	0.85	0.86
PR alone	0.86	0.86	0.88	0.89

HCV indicates hepatitis C virus; N/A, not applicable; PR, peginterferon alfa-2a plus ribavirin; SVR, sustained virologic response; TVR, telaprevir.

a All parameter estimates are derived from ADVANCE and REALIZE [13], [14], [15] (Vertex Pharmaceuticals, unpublished data, 2011).

b Fibrosis score distributions reflect the combined patient populations of 12-week TVR+PR and PR-alone treatment arms of the clinical trials. Values in each column do not sum to 100% due to rounding; actual values sum to 100%.

c Data from ADVANCE and REALIZE were available for patients with baseline fibrosis scores F0 and F1 combined. The model assumed half had baseline fibrosis score F0 and half had baseline fibrosis score F1.

d The model included severe treatment-related adverse events that occurred in 2% or more of patients in at least one treatment arm of ADVANCE and REALIZE. Incidence of anemia included moderately severe cases. From REALIZE, data were available for all previously treated patients combined.

e See Table S2 in Appendix S1 for utility scores from ADVANCE and REALIZE, from which average utility values were calculated.

**Table 2:** Input Parameter Values, by Health State, Age, and Sex.

Input Parameter	Male <50 Years	Male ≥50 Years	Female <50 Years	Female ≥50 Years
**Annual transition probabilities between METAVIR health states, no SVR** a [**4**]				
F0 to F1	0.1550	0.1938	0.0550	0.0688
F1 to F2	0.1058	0.1323	0.0510	0.0714
F2 to F3	0.1506	0.1883	0.0700	0.0875
F3 to F4	0.1577	0.1971	0.0480	0.0600
**Annual transition probabilities to serious liver disease, liver transplant, or death**				
F3 with SVR to HCC [21]	0.001			
F3 without SVR to HCC [21]	0.001			
F4 with SVR to HCC [22]	0.008			
F4 without SVR to HCC [22]	0.027			
F4 with SVR to DCC [22]	0.001			
F4 without SVR to DCC [22]	0.031			
DCC to HCC [23]	0.014			
DCC to liver transplant [21]	0.031			
DCC to death [23]	0.130			
HCC to liver transplant [24], [25]	0.060			
HCC to death [23]	0.430			
Liver transplant (year 1) to death [21]	0.210			
Liver transplant (year 2+) to death [21]	0.057			
**Post-treatment phase utility values ** [**26**]				
F0 to F4 with SVR	0.87			
F0 to F3 without SVR	0.81			
F4 without SVR	0.76			
DCC	0.69			
HCC	0.67			
Liver transplant, all years	0.77			
**Weekly HCV-treatment drug costs (wholesale acquisition cost)** b [**27**]				
Telaprevir (750 mg 3 times per day)	$5,039.24			
Peginterferon alfa-2a (180 µg/mL per week)	$673.65			
Ribavirin (weight-based dosing; cost based on 1,200 mg per day)	$99.19			
**Annual direct costs of HCV (chronic care) (in 2012 US dollars)**				
On-treatment (METAVIR F0–F4) [19], [28], [29]	$850.25			
Post-treatment, SVR not achieved (METAVIR F0–F4) [30]	$2,328.41			
Post-treatment, SVR achieved (METAVIR F0–F4)^c^	$0			
DCC [31]	$30,790			
HCC [31]	$48,290			
Liver transplant (year 1) [31]	$186,482			
Liver transplant (year 2+) [31]	$42,036			
**Weekly adverse-event costs (in 2012 US dollars)** [**28**] **,** [**29**] **,** [**32**] **,** [**33**]				
Anemia^d^	$38.59			
Fatigue	$35.23			
Headache	$35.23			
Leukopenia	$40.74			
Neutropenia	$40.74			
Rash	$35.23			

DCC indicates decompensated cirrhosis; HCC, hepatocellular carcinoma; HCV, hepatitis C virus; PR, peginterferon alfa-2a plus ribavirin; SVR, sustained virologic response; TVR, telaprevir; US, United States.

a Annual probabilities of progression in METAVIR fibrosis score for patients with SVR and with no or mild fibrosis (F0–F2) were assumed to be zero, which was consistent with data reported in various published studies [18], [34], [35], [36].

b Patients in the TVR+PR arm of the model received TVR for a total of 12 weeks and peginterferon alfa-2a plus ribavirin for a total of 24 or 48 weeks (see Figure 1).

c Post-treatment costs for patients who did not achieve SVR represent incremental costs over those incurred by patients who achieved SVR. Therefore, the model assumed post-treatment costs for patients who achieved SVR were $0. These post-treatment costs do not include additional costs incurred by patients who progressed to DCC, HCC, or liver transplant, which are shown separately.

d Consistent with the clinical trial protocols, anemia was managed with ribavirin dose reductions; therefore, costs of epoetin alfa were excluded from the analysis.

### Baseline Population Characteristics

Characteristics of the modeled population were assumed to be equivalent to those of patients in ADVANCE and REALIZE (Table 1) [13], [14], [15] (Vertex Pharmaceuticals, unpublished data, 2011).

### Clinical Efficacy

The primary efficacy measure used in the model was achievement of SVR. SVR rates were stratified by baseline METAVIR fibrosis (no or mild fibrosis [F0–F1, F2] and advanced fibrosis [F3, F4]) and obtained from ADVANCE and REALIZE (Table 1) [15] (Vertex Pharmaceuticals, unpublished data, 2011).

### Adverse-Event Incidence and Duration

The model accounted for the cost and quality-of-life implications of clinically relevant treatment-related adverse events. Incidence and duration were taken from ADVANCE and REALIZE (Table 1) [13], [14], [15] (Vertex Pharmaceuticals, unpublished data, 2011).

### Transition Probabilities for the Markov Model

Annual health-state transition probabilities were derived from published HCV economic models and epidemiology studies of disease progression with and without treatment (Table 2). The supplemental appendix further describes the sources of the individual transition probabilities (Appendix S1).

### Mortality

In any 1-year model cycle, patients in the HCC, DCC, and liver transplant health states were at risk of death from HCV-related causes [21], [23] (Table 2). Annual probabilities of death from all other causes, which could occur from any health state, were based on age- and sex-specific US general population mortality data [37].

### Resource Use and Costs

Costs of HCV treatment with TVR, peginterferon alfa-2a, and ribavirin were computed using drug unit costs, indicated dosing, and drug use observed in ADVANCE and REALIZE. Trial drug use captured all reasons for discontinuation, including eRVR, treatment futility, and adverse events (Table S1 in Appendix S1). Costs of care for HCV patients while on treatment were estimated using a microcosting approach, combining frequency of physician visits and lab tests specified in the American Association for the Study of Liver Diseases guidelines [19] and unit costs from Medicare reimbursement rates [28], [29].

Weekly costs of treatment-related adverse events were estimated using a microcosting approach with data from published treatment guidelines [32], [33] and Medicare reimbursement rates [28], [29].

Annual costs of post-treatment HCV management and annual costs of liver disease and associated sequelae (i.e., DCC, HCC, and liver transplant) were derived from separate retrospective analyses of managed care administrative claims data [30], [31]. Both studies included inpatient stays, outpatient visits, and pharmacy costs.

All costs are presented in 2012 US dollars (USD), except drug costs which are reported in 2013 USD (Table 2); where necessary, costs were inflated using the medical care component of the US consumer price index [38].

### Utility Values

Utility values quantify individual well-being on a scale from 0 (worst possible health or death) to 1 (perfect health) and were used to convert time spent on treatment and in each health state into estimates of quality-adjusted life-years (QALYs). For the treatment phase, mean utility values by treatment and patient subgroup were calculated by applying US-specific valuation weights to response data from the EQ-5D-3L [39] administered in ADVANCE and REALIZE [40] (Vertex Pharmaceuticals, unpublished data, 2011). Utility estimates implicitly accounted for quality-of-life decrements from treatment-related adverse events (Table 1; Table S2 in Appendix S1). In the post-treatment phase, health-state utility values were taken from a systematic review that translated SF-36 Health Survey data into community-weighted utilities [26] (Table 2).

### Model Outcomes

Health and economic outcomes derived from the model for both treatment arms included life-years, QALYs, cases of liver-disease complications, deaths, and total direct medical costs by type and overall. Additionally, the model used differences in these outcomes between the treatment arms to derive incremental cost-effectiveness ratios (ICERs), including the incremental cost per QALY gained. All outcomes are reported by patient subgroup and were discounted at an annual rate of 3% unless otherwise noted [41].

### Sensitivity Analyses

Sensitivity analyses were performed to assess the robustness of the model results and to determine the impact of parameter uncertainty. In one-way sensitivity analyses, input parameter values were varied individually across realistic ranges; in probabilistic sensitivity analyses (PSAs), all input parameter values were sampled from appropriate probability distributions and varied simultaneously in 10,000 Monte Carlo simulation runs. SVR rates were varied across estimated 95% confidence limits, assuming clinical trial values were beta-distributed. Because uncertainty information for other input parameters (i.e., chronic HCV costs by health state, adverse event costs, utilities, and health-state transition probabilities) was limited, the model utilized triangle distributions (in the PSAs) and relatively wide ranges (in the one-way sensitivity analyses). Scenario analyses also were performed to determine the impact of changes in model assumptions (e.g., discount rates and model time horizon) and to assess the cost-effectiveness of TVR+PR in various patient subpopulations (e.g., those based on sex, age, and starting METAVIR fibrosis score). All sensitivity analyses were performed on the model's primary cost-effectiveness outcome, incremental cost per QALY gained.

## Results

### Base-Case Analysis

Table 3 displays the average per-patient lifetime results of the model by patient subgroup. The model predicted that patients receiving TVR+PR were less likely to experience compensated cirrhosis, DCC, HCC, or liver transplant and were less likely to die from HCV-related causes than patients who received PR alone. Regardless of treatment history, patients treated with TVR+PR were estimated to have longer life expectancies (0.8–2.0 more life-years, discounted) and more QALYs (1.0–2.5 more QALYs, discounted) than patients treated with PR alone.

**Table 3:** Base-Case Model Results: Average Per-Patient Lifetime Health and Cost Outcomes by Patient Subgroup.

Outcomes	Treatment-Naïve Patients		Prior Relapsers		Prior Partial Responders		Prior Null Responders	
	TVR+PR	PR Alone	TVR+PR	PR Alone	TVR+PR	PR Alone	TVR+PR	PR Alone
**Health outcomes, discounted (undiscounted)**								
Life-years	20.3 (33.1)	19.3 (30.8)	18.7 (29.3)	16.6 (24.6)	17.4 (26.7)	16.3 (24.0)	16.5 (25.6)	15.5 (22.4)
QALYs	17.3 (28.3)	16.1 (25.5)	16.1 (25.2)	13.4 (19.8)	14.6 (22.3)	13.1 (19.2)	13.4 (20.0)	12.2 (17.5)
**Percentage with liver-disease complications and HCV-related death, undiscounted**								
Cirrhosis^a^	17.1%	41.9%	9.3%	50.9%	18.1%	48.8%	36.5%	60.5%
DCC	6.9%	16.3%	4.9%	25.2%	15.0%	26.6%	25.1%	33.8%
HCC	7.8%	16.1%	8.5%	24.1%	16.3%	26.2%	24.8%	32.4%
Liver transplant	1.4%	3.28%	1.2%	4.8%	3.0%	5.1%	4.9%	6.5%
HCV-related death	12.2%	26.5%	11.1%	39.7%	25.3%	42.4%	40.5%	53.8%
**Discounted costs and ICERs** b								
HCV-treatment drug costs	$77,293	$28,747	$77,946	$30,734	$89,122	$19,307	$80,013	$16,501
Adverse-event costs	$158	$161	$494	$336	$494	$336	$494	$336
Other direct medical costs	$21,686	$48,742	$17,706	$72,795	$44,021	$77,471	$71,183	$95,187
Total direct costs	$99,137	$77,650	$96,145	$103,865	$133,637	$97,114	$151,690	$112,024
Incremental cost per life-year gained (TVR+PR vs. PR)	$23,054	—	Dominates^c^	—	$31,528	—	$41,990	—
Incremental cost per QALY gained (TVR+PR vs. PR)	$16,778	—	Dominates^c^	—	$24,173	—	$34,279	—

DCC indicates decompensated cirrhosis; HCC, hepatocellular carcinoma; HCV, hepatitis C virus; ICER, incremental cost-effectiveness ratio; PR, peginterferon alfa-2a plus ribavirin; QALY, quality-adjusted life-year; TVR, telaprevir.

a Modeled cases of cirrhosis that developed following treatment.

b Due to rounding, ICERs differ slightly from calculations using costs, life-years, and QALYs shown in this table.

c One treatment dominates another if it exhibits more QALYs at a lower total cost.

Although the model projected higher HCV-treatment drug costs for patients who received TVR+PR than for patients who received PR alone, these costs were offset completely (for prior relapsers) or partially (for all other subgroups) by lower medical costs associated with chronic HCV care, liver disease, and its sequelae.

The calculation of ICERs showed that TVR+PR dominated PR alone (i.e., TVR+PR exhibited more QALYs at a lower cost than PR alone) in prior relapsers and exhibited incremental costs per QALY gained of $16,778 for treatment-naïve patients, $24,173 for prior partial responders, and $34,279 for prior null responders at the full acquisition costs of the products.

### Sensitivity Analyses

The one-way sensitivity analyses showed that the incremental cost per QALY gained for TVR+PR compared with PR alone remained below $40,000 for treatment-naïve patients and below $50,000 for most input parameter values tested for prior partial responders and prior null responders; TVR+PR remained dominant for prior relapsers for most input parameter values tested. Model results were most sensitive to changes in the base-case SVR rates and transition probabilities for disease progression (see tornado diagrams in Figure S1 in Appendix S1).

Cost-effectiveness acceptability curves generated by the PSAs showed that TVR+PR was likely to be cost-effective over a wide range of willingness-to-pay thresholds. At a threshold of $50,000 per QALY gained, the likelihood that TVR+PR was cost-effective exceeded 90% for all four patient subgroups (see cost-effectiveness acceptability curves in Figure S2 in Appendix S1).

Table 4 presents the results of scenario analyses. Model results were sensitive to patient characteristics (i.e., sex, age, and baseline METAVIR fibrosis score), discount rate, and shorter time horizons. However, ICERs remained around $60,000 per QALY gained or below, except when shorter time horizons were tested. For all patient subgroups, ICERs were generally lower among men than among women and lower among patients beginning HCV treatment before age 50 years than at or after age 50 years. Patients beginning HCV treatment with no or mild fibrosis had higher ICERs in some subgroups (treatment-naïve patients and prior relapsers) and lower ICERs in other subgroups (prior partial responders and prior null responders) compared with patients starting treatment with advanced fibrosis. Results were relatively insensitive to base-case assumptions regarding the probability of further liver deterioration after achievement of SVR and regarding treatment-phase utility values for TVR+PR and PR alone. However, assuming a utility value of 1.0 for all patients who achieved SVR resulted in lower ICERs than in the base case.

**Table 4:** Impact on Incremental Cost per QALY Gained of Starting Age, METAVIR Score, Sex, and Alternative Modeling Assumptions.

Scenario	Treatment-Naïve Patients	Prior Relapsers	Prior Partial Responders	Prior Null Responders
**Base-case analysis** a	$16,778	Dominates^b^	$24,173	$34,279
**Subgroups by sex**				
Males	$14,592	Dominates^b^	$149,806^c^	$29,548
Females	$25,100	$2,026	$24,381	$59,812
**Subgroups by age**				
Age ≥50 years	$21,795	Dominates^b^	$25,390	$31,756
Age <50 years	$14,229	Dominates^b^	$19,358	$39,359
**Subgroups by fibrosis score**				
No or mild fibrosis (F0–F2)	$19,172	$2,584	$20,965	$26,140
Advanced fibrosis (F3–F4)	$10,997	Dominates^b^	$26,861	$40,631
**Shorter time horizons**				
10 years	$210,415	$59,344	$274,035	$392,877
20 years	$66,980	$7,254	$74,294	$100,953
**Alternative annual discount rates**				
0%	Dominates^b^	Dominates^b^	$4,228	$9,505
5%	$34,919	$3,052	$44,890	$60,289
**Alternative utility values**				
Equal treatment-phase utility values for TVR+PR and PR alone	$16,597	Dominates^b^	$23,967	$33,953
Utility value of 1.0 for SVR	$10,175	Dominates^b^	$14,735	$22,074
**Alternative transition probability values**				
No liver deterioration after SVR regardless of METAVIR fibrosis score	$15,797	Dominates^b^	$22,148	$32,608
Lower probability of death after liver transplant (0.169 first year, 0.034 years 2+)^d^	$16,466	Dominates^b^	$23,908	$34,087

ICER indicates incremental cost-effectiveness ratio; PR, peginterferon alfa-2a plus ribavirin; QALY, quality-adjusted life-year; SVR, sustained virologic response; TVR, telaprevir.

a The base-case values and assumptions were as follows: patient population distribution by sex, age, and METAVIR fibrosis score from the ADVANCE and REALIZE clinical trials; lifetime time horizon; 3% annual discount rate; incremental difference in treatment-phase utility values for TVR+PR compared with PR alone of 0.01 for treatment-naïve patients and prior relapsers and –0.03 for prior partial responders and prior null responders; utility value of 0.87 for health states F0 to F4 if SVR was achieved; and clinical assumption that patients with advanced fibrosis (F3-F4) remained at risk for further liver deterioration even if SVR is achieved; probability of death after liver transplant of 0.210 in first year, 0.057 in years 2+.

b TVR+PR dominates PR alone, with more QALYs at a lower total cost.

c The subgroup of male prior partial responders in the PR arm of REALIZE was very small (n = 15). The high ICER for this subgroup was the result of the SVR achieved by the 1 male with baseline fibrosis score F4. Caution should be exercised in interpreting this ICER.

d Based on estimates reported in Thein et al., 2009 [42].

## Discussion

The economic model projected that higher SVR rates observed in ADVANCE and REALIZE for TVR+PR, compared with PR alone, led to fewer HCV-related complications and deaths and to increased survival and quality-adjusted survival in all patient subgroups examined in the analysis. In addition, fewer costly liver disease complications resulted in reductions in lifetime HCV-related costs. Model results demonstrated that TVR+PR was cost saving (dominant) in prior relapsers and cost-effective in all other patient subgroups at commonly cited willingness-to-pay thresholds in the US (i.e., thresholds of $50,000 per QALY gained or higher) [43]. These results are based on a US payer perspective, including only direct medical costs. Indirect costs, such as work and productivity losses, were not included in the model. However, the inclusion of such indirect costs would likely favor TVR+PR (lowering ICERs), assuming indirect costs are lower in patients with shorter treatment duration, in patients with SVR, and in patients with fewer HCV-related complications.

Sensitivity analyses indicated that model results were robust to input parameter uncertainty. Similar to other models, scenario analyses found that our cost-effectiveness results stratified by sex, age, and starting METAVIR fibrosis score showed substantial differences [44], [45]. In general, treatment with TVR+PR was most cost-effective in subpopulations that, without a curative treatment, would have experienced the greatest number of liver-disease complications over their remaining lifetimes (i.e., men and patients starting treatment before age 50 years). However, TVR+PR remained cost-effective at commonly cited willingness-to-pay thresholds in all patient subgroups, regardless of sex (see Table 4 for footnote regarding male prior partial responders), age, or starting METAVIR fibrosis score.

Cost-effectiveness results also were sensitive to the model time horizon. The cost of HCV treatment is incurred in the near term, and while achieving SVR has short-term benefits [30], [46], the full benefit of treatment is realized over the remaining patient lifetime. Shorter time horizons thus account for the full cost of treatment but not the full benefit. For this reason, the lifetime time horizon is recommended for cost-effectiveness analyses, especially for curative treatments of chronic diseases [47].

A few studies have assessed the cost-effectiveness of PR alone in treatment-naïve patients. To validate the accuracy of our model, we compared results from our PR-alone arm to the corresponding results from these published studies. For treatment-naïve patients with genotype 1 HCV infection treated with PR alone, our model's estimated life expectancy, quality-adjusted life expectancy, and discounted lifetime direct medical costs (30.8 undiscounted life-years, 16.1 discounted QALYs, and $77,650, respectively) were similar to those reported in previous US models for similar populations (approximately 30 undiscounted life-years, 19.4 discounted QALYs, and $58,179 in 2012 US dollars) [44], [48], [49]. However, a newer cost-effectiveness analysis estimated substantially lower QALYs (8.84-10.97 discounted QALYs) and much higher discounted lifetime costs (approximately $160,000) [50] when compared with our model and other published studies [44], [48], [49], [51]. These discrepancies may be attributable to different input parameter values for patient characteristics, transition probabilities, SVR rates, utility values, disease-related costs, and discontinuation rates, but specific drivers of the discrepant results were not readily apparent.

The cost-effectiveness ratios estimated by the model for TVR+PR versus PR alone were within the range reported for peginterferon plus ribavirin versus interferon plus ribavirin for patients with genotype 1 HCV infection (range: $2,600–$35,000) [44], [48], [49], [51]. A recent cost-effectiveness analysis of TVR+PR versus PR alone reported higher ICERs ($47,400 and $91,000 for patients with advanced and mild fibrosis, respectively) [50] than our model, but the comparison is problematic because of highly discrepant input parameter values. Specifically, the authors developed a model based on the input values from a “general protease inhibitor” that consisted of pooled effectiveness data for TVR+PR and a competitive combination therapy [50]. Results from our model for TVR+PR compared favorably with ICERs for other established health care interventions, such as antiviral drugs for hepatitis B virus infection ($27,184 for entecavir vs. no treatment) [52] and human immunodeficiency virus infection ($23,057 for darunavir/r to $69,500 for enfuvirtide vs. alternative regimens) [53], [54].

An important strength of our study is that we avoided some of the simplifying assumptions made in previous HCV models, which have been shown to bias cost-effectiveness results in favor of HCV treatment. These assumptions included assigning a utility value of 1.0 and assuming no further disease progression or liver-disease complications for patients achieving SVR [55]. We assigned a utility value of 0.87 to patients after achievement of SVR, and allowed patients with advanced fibrosis to continue to progress and develop liver-disease complications (but with lower risk than those not achieving SVR). These conservative assumptions are likely to more realistically reflect quality of life for patients with prior HCV diagnosis (in terms of age and comorbidities) and continued disease progression for treated patients with advanced fibrosis [21], [22].

Several limitations of this analysis should be mentioned. As with any economic model, the accuracy of the results depends on the robustness of the data inputs and assumptions. Long-term data on the natural progression of HCV infection and the impact of SVR are limited because HCV infection progresses slowly. The best available data were used, including a long-term observational study that followed 384 patients with HCV-related cirrhosis up to 12 years [56] and a systematic review, meta-analysis, and meta-regression of 111 published studies of disease progression from the stages of fibrosis to cirrhosis [5]. In addition, we used health state–specific utility and cost estimates rather than age-specific values due to limitations in the available data for these estimates. The impact of this data limitation on the results is difficult to predict because of the interplay between the various parameters in the model.

Another limitation of our study was the use of efficacy and drug-use inputs from two Phase III clinical trials, which represented controlled environments rather than real-world conditions. However, efficacy and drug costs in the model did account for discontinuation due to eRVR, treatment futility, and all other reasons, in accordance with the recommended clinical use of the treatment regimens. As a result, average per-patient treatment costs in our model ($77,293-$89,122, depending on patient subgroup) were substantially lower than the cost of a full course of TVR+PR therapy ($97,567). Persistency and its relationship to treatment effectiveness in real-world settings is the subject of ongoing studies.

Characteristics of our modeled population also were based on those of participants in the two Phase III clinical trials (i.e., patients with genotype 1 HCV monoinfection). Further, our analysis for previously treated patients considered prior therapy with pegylated interferon (alfa-2a or alfa-2b) plus ribavirin only (non-pegylated interferon was excluded), consistent with REALIZE. Thus, the model's results may not be generalizable to all US HCV patients seeking treatment. However, because genotype 1 accounts for about 75% of all cases of HCV infection in the US [8] (Nainan et al., 2006) and because treatment with peginterferon plus ribavirin has been the standard-of-care treatment for almost a decade, the cost-effectiveness results of our model are widely relevant. Moreover, age and sex distributions in ADVANCE and REALIZE are consistent with a recent study of HCV prevalence in the US [2].

Although our model investigated the cost-effectiveness of TVR+PR compared with PR alone in a variety of interesting subgroups based on level of treatment experience, age, sex, and initial fibrosis score, there are additional subgroups that may merit further investigation. For example, race has been shown to be associated with SVR, and ADVANCE demonstrated a significant difference in SVR for TVR+PR versus PR alone (62% vs. 25%) among black participants [13]. Additionally, the IL28B CC genotype has been shown to be associated with increased treatment efficacy for TVR+PR and PR alone in both treatment-naïve and treatment-experienced patients. Although not the focus of this study, a recent analysis using our model estimated the cost-effectiveness of initial TVR+PR treatment compared with TVR+PR re-treatment after PR alone, if necessary. When individuals failing initial PR-alone therapy were assumed to be re-treated with TVR+PR at rates of 50% to 75%, ICERs ranged from $25,517 to $30,766 per QALY gained, respectively [57].

Finally, our analysis focused on TVR+PR compared with PR alone and did not consider other comparisons. Our analysis was based on head-to-head data from Phase III, randomized, double-blinded, multicenter trials in treatment-naïve patients (ADVANCE) and treatment-experienced patients (REALIZE) with chronic genotype 1 HCV infection [13], [14]. Comparisons with boceprevir and newer HCV treatments, such as simeprevir and sofosbuvir, represent important future work. Boceprevir and simeprevir are both protease inhibitors approved for use in patients with genotype 1 HCV infection; sofosbuvir is a NS5B nucleotide polymerase inhibitor approved for use in patients with genotype 1, 2, 3, or 4 HCV infection. No head-to-head data are currently available for any of these comparisons. Meta-analysis results are available only for comparison with boceprevir; however, several available meta-analyses have shown highly discrepant results [58]. Future work is needed to help decision makers understand the comparative efficacy, safety, and cost-effectiveness of TVR+PR compared with all existing options for HCV treatment.

## Conclusion

This decision-analytic model estimated that the higher SVR rates observed for TVR+PR, compared with PR alone, led to fewer HCV-related complications and deaths as well as increased survival and quality-adjusted survival in all patient subgroups. Within the limitations of our model, TVR+PR was projected to be a cost-effective or cost-saving strategy compared with PR for the treatment of adults with chronic genotype 1 HCV infection and compensated liver disease from a US payer perspective. Additionally, our model projected that higher HCV-treatment drug costs for patients receiving TVR+PR compared with PR alone would be offset largely or completely, depending on patient subgroup, by reductions in medical costs associated with liver-disease complications. Future analyses are needed to compare TVR+PR with all existing HCV treatment options.

## Supporting Information

Appendix S1 **Additional Information, Tables, and Figures.**
(PDF)
