# Supplementary material for: Cost-effectiveness of Telaprevir Combination Therapy for Chronic Hepatitis C
Source: PLoS One. 2014 Mar 6;9(3):e90295. doi: 10.1371/journal.pone.0090295 (PMC3946047; doi:10.1371/journal.pone.0090295)
Supplement: Appendix S1 — Additional Information, Tables, and Figures. (PDF) [file pone.0090295.s001.pdf]

Appendix S1. Additional Information, Tables, and Figures

Appendix to: Cost-effectiveness of Telaprevir Combination Therapy for Chronic Hepatitis C Virus Infection in the United States

Authors: Anita J. Brogan, PhD; Sandra E. Talbird, MSPH; James R. Thompson, MS; Jeffrey D. Miller, MS; Jaime Rubin, MPH; Baris Deniz, MSc

This appendix provides additional details on the health-state transition probabilities, the percentages of participants in each trial arm who received the components of their assigned regimens over the course of the treatment period in the ADVANCE and REALIZE clinical trials, the derivation of the utility values used in the model, and the results of the one-way and probabilistic sensitivity analyses.

Additional Information on Transition Probabilities Used in the Post-Treatment Phase of the Model

Annual probabilities of progression in METAVIR fibrosis score for patients not achieving sustained virologic response (SVR), stratified by sex and age (< 50 years, ≥ 50 years), were obtained from a recent disease progression model (Davis et al., 2010) and based on a meta-analysis of 111 published studies on hepatitis C virus (HCV) disease progression (Thein et al., 2008). Annual probabilities of progression in METAVIR fibrosis score for patients with SVR and with no or mild fibrosis (F0-F2) were assumed to be zero, which was consistent with data reported in various published studies (Poynard et al., 2000; Poynard et al., 2002; Cammà et al., 2004; Sherman et al., 2011).

Transition probabilities between the other health states (METAVIR fibrosis scores F3 and F4, decompensated cirrhosis [DCC], hepatocellular carcinoma [HCC], liver transplant, and HCV-related death) were derived from a comprehensive review of the published literature, including published HCV economic models and epidemiology studies of disease progression with and without treatment (Ascher et al., 1994; Bennett al., 1997; Berenguer et al., 2009; Bruno et al., 2007; Detre et al., 1996; Di Marco et al., 2007; Fattovich et al., 1997; Gramenzi et al., 2001; Grieve and Roberts, 2002; Grieve et al., 2006; Kilpe et al.,1993; Okuda et al., 1985; Saab et al., 2010; Salomon et al., 2003; Shepherd et al., 2007; Siebert et al., 2003; Sullivan et al., 2004; Thein et al., 2008; Thuluvath et al., 2010; Veldt et al., 2007; Wong and Koff, 2000). Specifically, values for the transition from F3 to HCC for patients with or without SVR were assumed to be the same and were taken from Bennett and colleagues' (1997) cost-effectiveness analysis. Values for transitions from compensated cirrhosis (F4) to either DCC or HCC differed by SVR status and were taken from Saab and colleagues' (2010) economic evaluation of the impact of antiviral

therapy at different stages of HCV infection. Saab and colleagues' estimates were used because these estimates were based on the published natural history studies and clinical trials that were analyzed in the meta-analysis and meta-regression performed by Thein and colleagues (2008). Because clinical trial data were included in the meta-analysis and meta-regression, Saab and colleagues (2010) were able to generate disease progression estimates that differentiated between patients with SVR and patients without SVR.

Transition probabilities from DCC to HCC and from DCC or HCC to death were based on Fattovich and colleagues' (1997) natural history study that followed 384 patients with compensated cirrhosis resulting from HCV infection. This study was used by Grieve and colleagues (2006) to estimate transition probabilities in a cost-effectiveness analysis comparing pegylated interferon with interferon treatment, both with ribavirin.

The probability of liver transplant was estimated by dividing the number of liver transplants performed each year in the United States due to DCC or HCC by the prevalent population with DCC or HCC, respectively. These estimates were obtained from Bennett and colleagues (1997) for DCC and from Thuluvath and colleagues (2010) and the National Cancer Institute's (2009) Surveillance, Epidemiology, and End Results (SEER) statistics for HCC. The probabilities of death in the first year and in subsequent years following a liver transplant were estimated from three observational studies of individuals undergoing liver transplant as a result of HCV infection (Ascher et al., 1994; Detre et al., 1996; Kilpe et al., 1993). These studies were used in several later cost-effectiveness analyses, including the analyses by Bennett and colleagues (1997) and Siebert and colleagues (2003).

Appendix Tables

Table S1. Drug Use Observed in ADVANCE and REALIZE by Patient Subgroup and 4-Week Time Interval[a]

Week Interval	Treatment-Naïve Patients			Prior Relapsers[b]			Prior Partial Responders			Prior Null Responders		
	TVR+PR		PR Alone	TVR+PR		PR Alone	TVR+PR		PR Alone	TVR+PR		PR Alone
	TVR	PR	PR	TVR	PR	PR	TVR	PR	PR	TVR	PR	PR
0-4	100.0%	100.0%	100.0%	100.0%	100.0%	100.0%	100.0%	100.0%	100.0%	100.0%	100.0%	100.0%
4-8	93.1%	95.9%	96.7%	95.1%	97.9%	98.5%	93.8%	99.0%	96.3%	96.6%	97.3%	100.0%
8-12	84.8%	93.4%	95.6%	90.2%	95.1%	95.6%	88.7%	94.8%	88.9%	70.7%	95.2%	97.3%
12-16	—	91.5%	94.7%	—	93.7%	95.6%	—	93.8%	88.9%	—	93.2%	97.3%
16-20	—	87.1%	83.4%	—	92.0%	89.7%	—	89.7%	59.3%	—	80.3%	29.7%
20-24	—	85.4%	82.5%	—	90.9%	89.7%	—	88.7%	59.3%	—	70.1%	29.7%
24-28	—	28.9%	81.4%	—	15.7%	86.8%	—	86.6%	51.9%	—	65.3%	27.0%
28-32	—	22.0%	61.5%	—	15.7%	75.0%	—	79.4%	25.9%	—	57.1%	16.2%
32-36	—	21.8%	59.8%	—	15.4%	66.2%	—	79.4%	22.2%	—	51.0%	10.8%
36-40	—	20.9%	59.0%	—	15.4%	64.7%	—	79.4%	18.5%	—	49.0%	10.8%
40-44	—	20.9%	58.4%	—	15.4%	61.8%	—	79.4%	18.5%	—	46.3%	10.8%
44-48	—	20.1%	56.8%	—	15.4%	61.8%	—	79.4%	14.8%	—	43.5%	10.8%

eRVR indicates extended rapid virologic response; HCV, hepatitis C virus; PR, pegylated interferon alfa-2a plus ribavirin; RGT, response-guided therapy; TVR, telaprevir.

[a] Captures all reasons for drug discontinuation, including eRVR, treatment futility, and adverse events. These data are used in the treatment phase of the model to calculate total HCV-treatment drug costs for each arm and to weight the treatment-phase utility scores by the amounts of time spent on TVR and PR, on PR only, or off treatment in each arm (Vertex Pharmaceuticals, unpublished data, 2011).

[b] In REALIZE, prior relapsers were not eligible for RGT (i.e., they were not allowed to stop PR therapy at week 24 if they achieved eRVR). However, the model utilized adjusted drug use data to account for RGT in this patient subgroup for consistency with the telaprevir prescribing information (INCIVEK, 2012).

Table S2. Utility Values, by Drug Use and Averaged Over the 72-Week Treatment Period

Data Type/ Regimen	Treatment-Naïve Patients	Previously Treated Patients		
		Prior Relapsers	Prior Partial Responders	Prior Null Responders
**Utility scores by drug use (converted from clinical trial EQ-5D data)[a]**				
TVR+PR				
On treatment with TVR and PR	0.798	0.798		
On treatment with PR	0.825	0.808		
Off treatment[b]	0.911	0.913		
PR alone				
On treatment with PR	0.818	0.826		
Off treatment[b]	0.911	0.913		
**Average utility values for 72-week treatment phase[c,d]**				
TVR+PR	0.876	0.873	0.850	0.862
PR alone	0.863	0.865	0.883	0.887

EQ-5D indicates 5-dimension EuroQol quality-of-life instrument; PR, pegylated interferon alfa-2a plus ribavirin; TVR, telaprevir.

[a] Utility scores for each treatment arm were converted from clinical trial EQ-5D data (Vertex Pharmaceuticals, unpublished data, 2011) and implicitly account for treatment-related adverse events. For the TVR+PR arm, utility scores were collected separately for two time periods: first, the time during which patients were on treatment with TVR and PR; second, the time during which patients were on treatment with PR only.

[b] Modeled patients who were off treatment for any reason (i.e., eRVR, treatment futility, adverse events, or any other reason) were assigned utility scores calculated from baseline (pre-treatment) EQ-5D data for participants in the pooled 12-week TVR+PR and PR-alone arms of the clinical trials.

[c] Calculated by multiplying the drug-specific utility scores above by the respective amounts of time spent on treatment with TVR and PR, on PR only, or off treatment in each patient subgroup (see Table S1). These average utility values are applied by the model over the 72-week treatment phase and account for patients who discontinued part or all of their HCV-treatment regimen during the treatment period.

[d] Due to the availability of response-guided therapy for treatment-naïve patients and prior relapsers treated with TVR+PR, the model estimated higher utility values, on average, for the TVR+PR arm than for the PR-alone arm in these patient subgroups. Response-guided therapy allowed patients in the TVR+PR arm to spend more time off therapy, when utility scores were higher.

Appendix Figures

Figure S1. One-Way Sensitivity Analysis Results: Tornado Diagrams

Figure S1a. Treatment-Naïve Patients

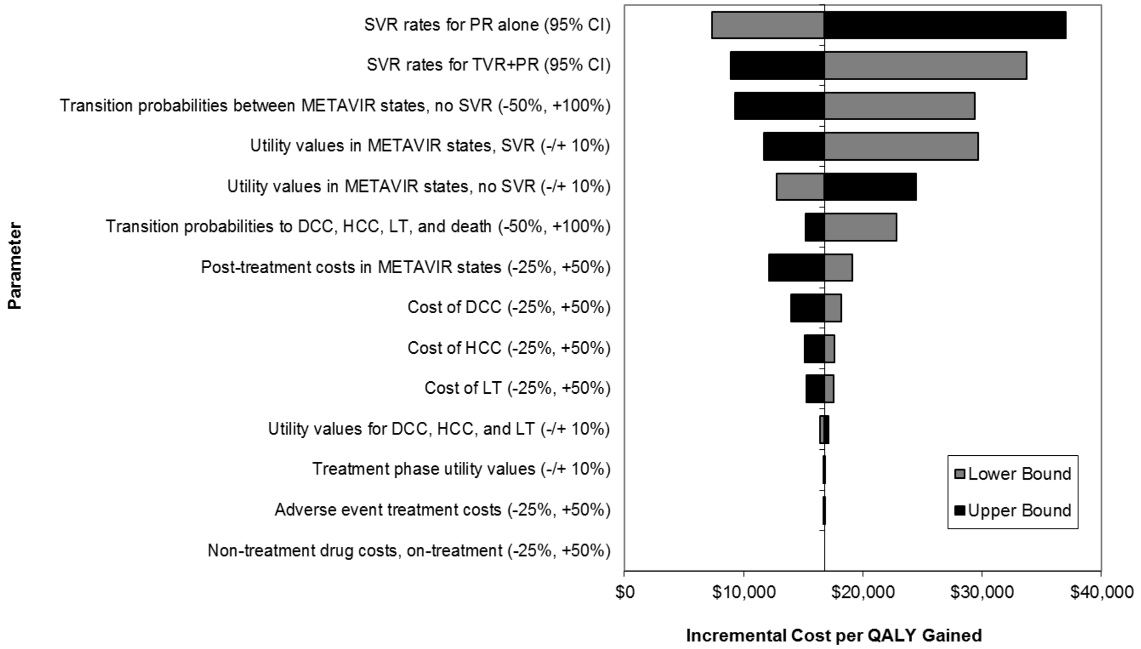

Figure S1b. Prior Relapsers[a]

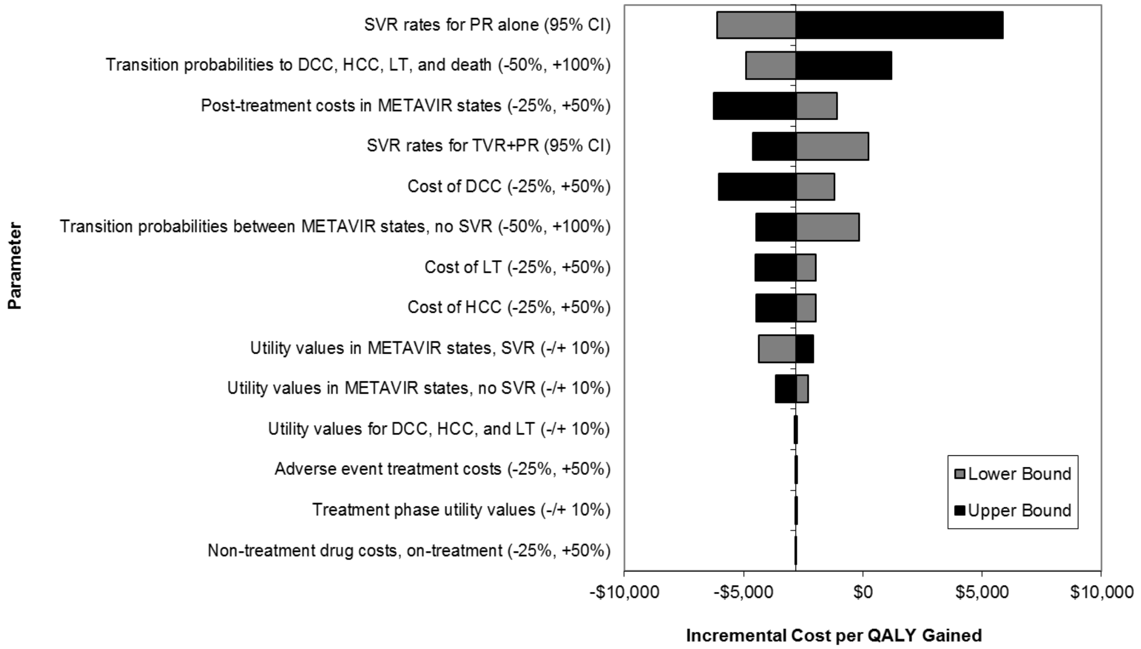

Figure S1c. Prior Partial Responders

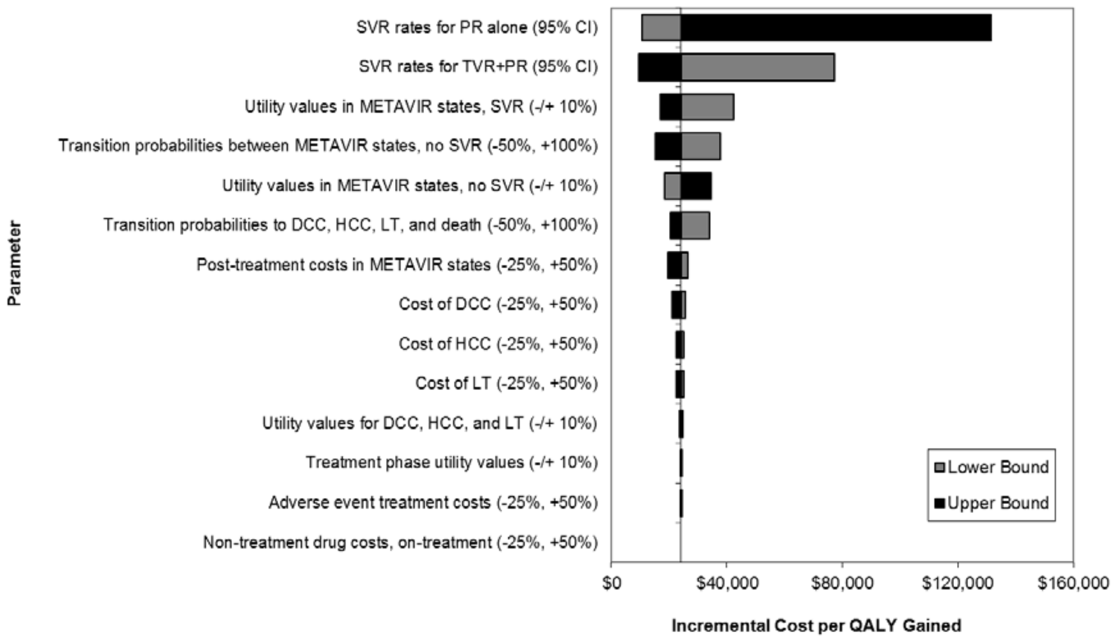

Figure S1d. Prior Null Responders

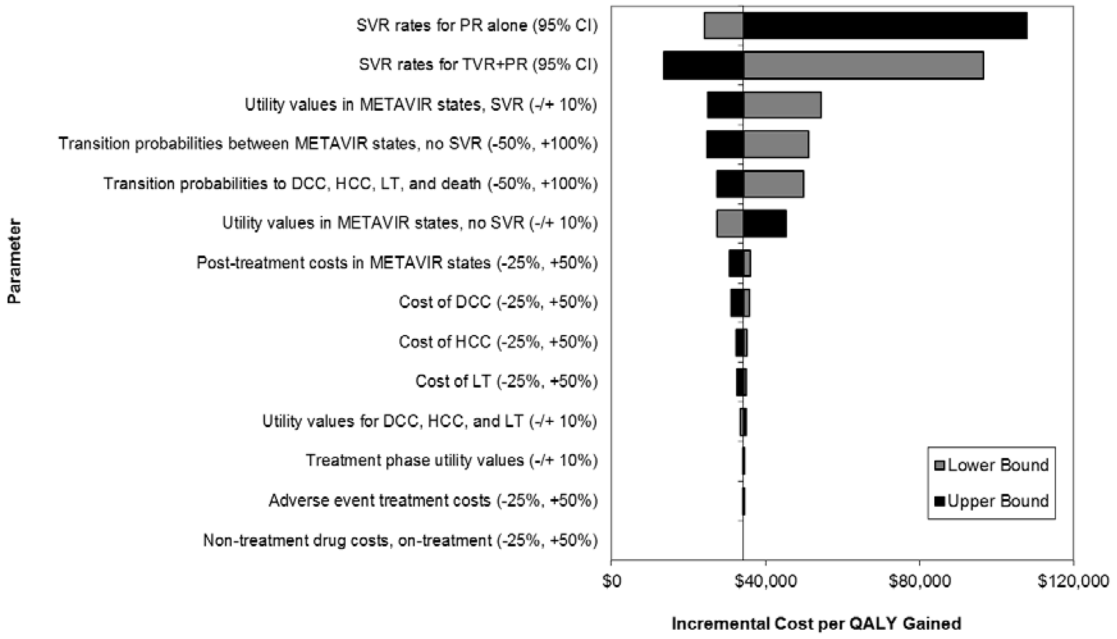

CI indicates confidence interval; DCC, decompensated cirrhosis; ICER, incremental cost-effectiveness ratio; HCC, hepatocellular carcinoma; LT, liver transplant; PR, pegylated interferon alfa-2a plus ribavirin; QALY, quality-adjusted life-year; SVR, sustained virologic response; TVR, telaprevir.

[a] Negative ICERs indicate that TVR+PR dominated PR alone (i.e., TVR+PR exhibited more QALYs at a lower total cost than PR alone).

Figure S2. Probabilistic Sensitivity Analysis Results: Cost-effectiveness Acceptability Curves

Figure S2a. Treatment-Naïve Patients

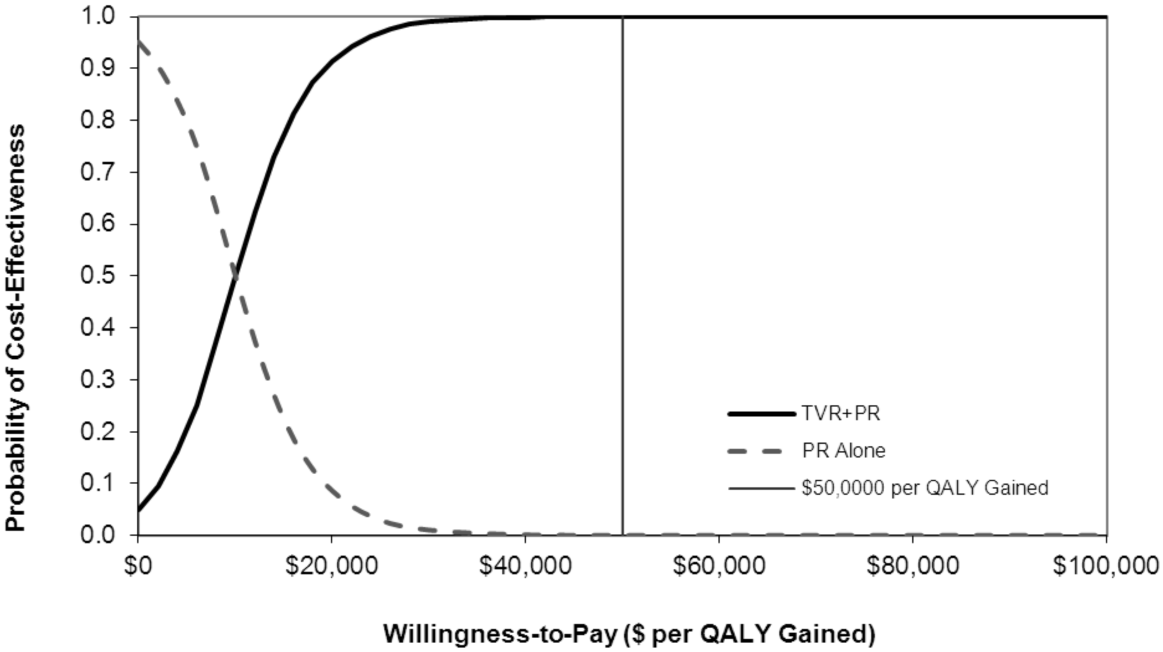

Figure S2b. Prior Relapsers[a]

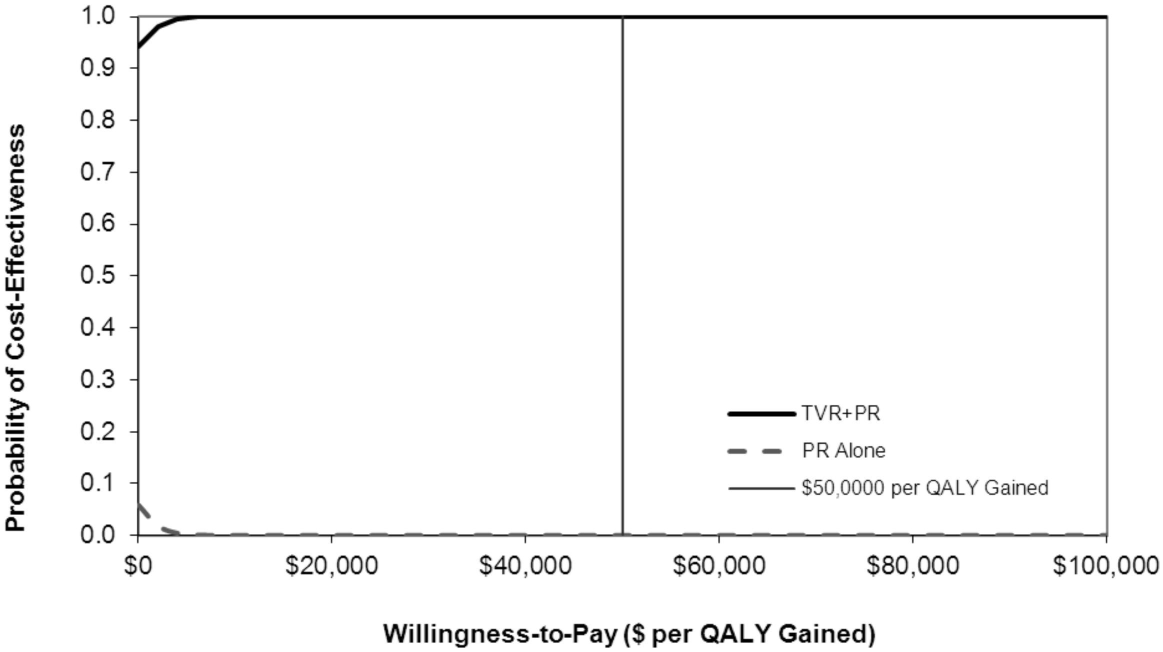

Figure S2c. Prior Partial Responders

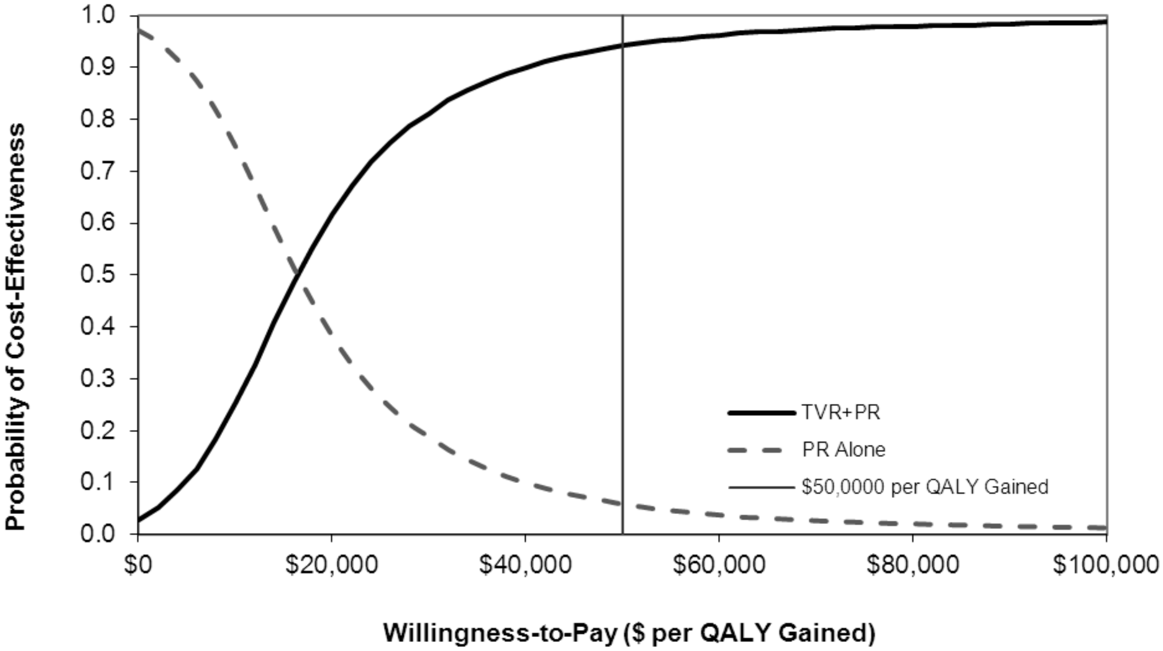

Figure S2d. Prior Null Responders

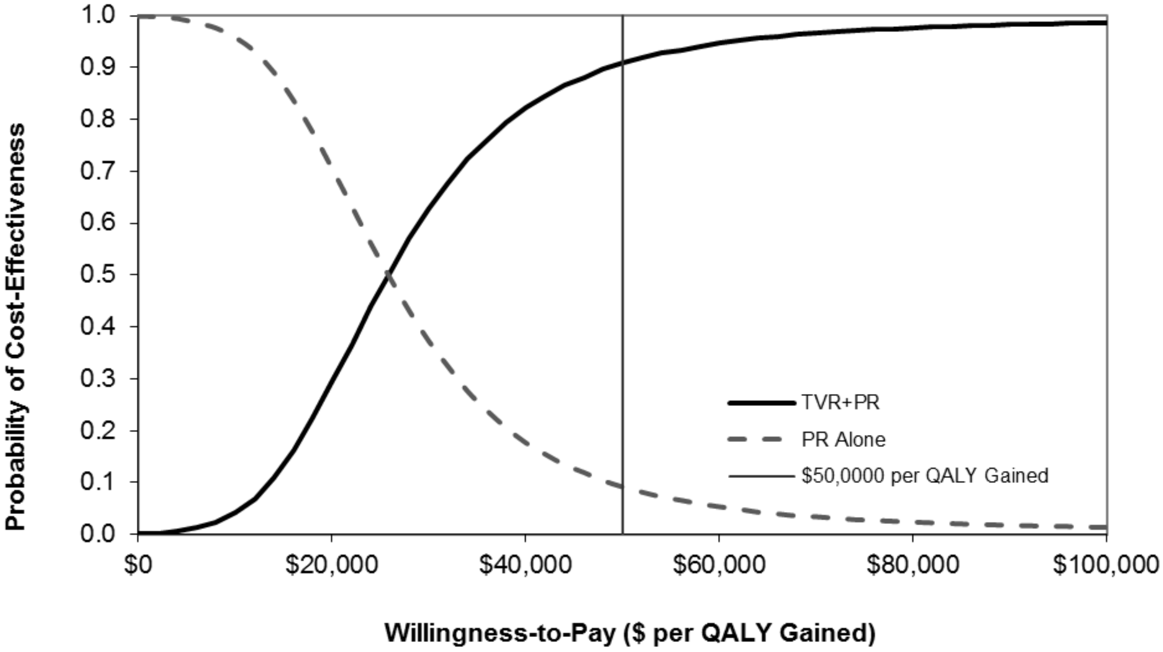

PR indicates pegylated interferon alfa-2a plus ribavirin; QALY, quality-adjusted life-year; TVR, telaprevir.

[a] Among prior relapsers, TVR+PR was the cost-effective choice in over 94% of simulation runs for all willingness-to-pay thresholds tested between $0 and $100,000 per QALY gained.

References

Ascher NL, Lake JR, Emond J, Roberts J (1994) Liver transplantation for hepatitis C virus-related cirrhosis. Hepatology 20 (1 pt 2): 24S-27S.

Bennett WG, Inoue Y, Beck JR, Wong JB, Pauker SG, et al. (1997) Estimates of the cost-effectiveness of a single course of interferon-alpha 2b in patients with histologically mild chronic hepatitis C. Ann Int Med 127: 855-865.

Berenguer J, Alvarez-Pellicer J, Martín PM, López-Aldeguer J, Von-Wichmann MA, et al.; GESIDA 3603/5607 Study Group (2009) Sustained virological response to interferon plus ribavirin reduces liver-related complications and mortality in patients coinfected with human immunodeficiency virus and hepatitis C virus. Hepatology 50: 407-413.

Bruno S, Stroffolini T, Colombo M, Bollani S, Benvegnù L, et al.; Italian Association of the Study of the Liver Disease (AISF) (2007) Sustained virological response to interferon-α is associated with improved outcome in HCV-related cirrhosis: a retrospective study. Hepatology 45: 579-587.

Cammà C, Di Bona D, Schepis F, Heathcote EJ, Zeuzem S, et al. (2004) Effect of peginterferon alfa-2a on liver histology in chronic hepatitis C: a meta-analysis of individual patient data. Hepatology 39: 333-342.

Davis GL, Alter MJ, El-Serag H, Poynard T, Jennings LW (2010) Aging of hepatitis C virus (HCV)-infected persons in the United States: a multiple cohort model of HCV prevalence and disease progression. Gastroenterology 138: 513-521, 521, e1-e6.

Detre KM, Belle SH, Lombardero M (1996) Liver transplantation for chronic viral hepatitis. Viral Hepatitis Reviews 2: 219-228.

Di Marco V, Almasio PL, Ferraro D, Calvaruso V, Alaimo G, et al. (2007) Peg-interferon alone or combined with ribavirin in HCV cirrhosis with portal hypertension: a randomized controlled trial. J Hepatology 47: 484-491.

Fattovich G, Giustina G, Degos F, Tremolada F, Diodati G, et al. (1997) Morbidity and mortality in compensated cirrhosis type C: a retrospective follow-up study of 384 patients. Gastroenterology 112: 463-472.

Gramenzi A, Andreone P, Fiorino S, Cammà C, Giunta M, et al. (2001) Impact of interferon therapy on the natural history of hepatitis C virus related cirrhosis. Gut 48: 843-848.

Grieve R, Roberts J, Wright M, Sweeting M, DeAngelis D, et al. (2006) Cost effectiveness of interferon alpha or peginterferon alpha with ribavirin for histologically mild chronic hepatitis C. Gut 55: 1332-1338.

Grieve R, Roberts J (2002) Economic evaluation for hepatitis C. Acta Gastroenterologica Belgica 65: 104-109.

INCIVEK™ (telaprevir) film coated tablets [prescribing information]. Cambridge, MA: Vertex Pharmaceuticals Incorporated; 2012.

Kilpe VE, Krakauer H, Wren RE (1993) An analysis of liver transplant experience from 37 transplant centers as reported to Medicare. Transplantation 56: 554-561.

National Cancer Institute (2009) Fast stats: an interactive tool for access to SEER cancer statistics. Liver and intrahepatic bile duct cancer; complete prevalence. SEER November 2009 submission. Available: http://seer.cancer.gov/faststats. Accessed 24 February 2011.

Okuda K, Ohtsuki T, Obata H, Tomimatsu M, Okazaki N, et al. (1985) Natural history of hepatocellular carcinoma and prognosis in relation to treatment. Study of 850 patients. Cancer 56: 918-928.

Poynard T, McHutchison J, Davis GL, Esteban-Mur R, Goodman Z, et al. (2000) Impact of interferon alfa-2b and ribavirin on progression of liver fibrosis in patients with chronic hepatitis C. Hepatology 32: 1131-1137.

Poynard T, McHutchison J, Manns M, Trepo C, Lindsay K, et al. (2002) Impact of pegylated interferon alfa-2b and ribavirin on liver fibrosis in patients with chronic hepatitis C. Gastroenterology 122: 1303-1313.

Saab S, Hunt DR, Stone MA, McClune A, Tong MJ (2010) Timing of hepatitis C antiviral therapy in patients with advanced liver disease: a decision analysis model. Liver Transplant 16: 748-759.

Salomon JA, Weinstein MC, Hammitt JK, Goldie SJ (2003) Cost-effectiveness of treatment for chronic hepatitis C infection in an evolving patient population. JAMA 290: 228-237.

Shepherd J, Jones J, Hartwell D, Davidson P, Price A, et al. (2007) Interferon alpha (pegylated and non-pegylated) and ribavirin for the treatment of mild chronic hepatitis C: a systematic review and economic evaluation. Health Technol Assess 11: 1-205, iii.

Sherman KE, Sulkowski MS, Zoulim F, et al. (2011) Follow-up on SVR durability and viral resistance in patients with chronic hepatitis C treated with telaprevir-based regimens: interim analysis of the EXTEND study. Hepatology 54: 485A-486A.

Siebert U, Sroczynski G, Rossol S, Wasem J, Ravens-Sieberer U, et al.; German Hepatitis C Model (GEHMO) Group; International Hepatitis Interventional Therapy (IHIT) Group (2003) Cost effectiveness of peginterferon alpha-2b plus ribavirin versus interferon alpha-2b plus ribavirin for initial treatment of chronic hepatitis C. Gut 52: 425-432.

Sullivan SD, Jensen DM, Bernstein DE, Hassanein TI, Foster GR, et al. (2004) Cost-effectiveness of combination peginterferon alpha-2a and ribavirin compared with interferon alpha-2b and ribavirin in patients with chronic hepatitis C. Am J Gastroenterol 99: 1490-1496.

Thein HH, Yi Q, Dore GJ, Krahn MD (2008) Estimate of stage-specific fibrosis progression rates in chronic hepatitis C virus infection: a meta-analysis and meta-regression. Hepatology 48: 418-431.

Thuluvath PJ, Guidinger MK, Fung JJ, Johnson LB, Rayhill SC, et al. (2010) Liver transplantation in the United States, 1999-2008. Am J Transplant 10 (4 pt 2): 1003-1019.

Veldt BJ, Heathcote J, Wedemeyer H, Reichen J, Hofmann WP, et al. (2007) Sustained virologic response and clinical outcomes in patients with chronic hepatitis C and advanced fibrosis. Ann Int Med 147: 677-684.

Wong JB, Koff RS (2000) Watchful waiting with periodic liver biopsy versus immediate empirical therapy for histologically mild chronic hepatitis C: a cost-effectiveness analysis. Ann Int Med 133: 665-675.
